# *AncestryPainter*: A Graphic Program for Displaying Ancestry Composition of Populations and Individuals

**DOI:** 10.1016/j.gpb.2018.05.002

**Published:** 2018-11-22

**Authors:** Qidi Feng, Dongsheng Lu, Shuhua Xu

**Affiliations:** 1 CAS Key Laboratory of Computational Biology, Max Planck Independent Research Group on Population Genomics, CAS-MPG Partner Institute for Computational Biology, Shanghai Institutes for Biological Sciences, Chinese Academy of Sciences, Shanghai 200031, China; 2 University of Chinese Academy of Sciences, Beijing 100049, China; 3 School of Life Science and Technology, ShanghaiTech University, Shanghai 201210, China; 4 Center for Excellence in Animal Evolution and Genetics, Chinese Academy of Sciences, Kunming 650223, China; 5 Collaborative Innovation Center of Genetics and Development, Shanghai 200438, China; 6 Human Phenome Institute, Fudan University, Shanghai 201203, China

**Keywords:** Graphic program, Admixed populations, Ancestry composition, *AncestryPainter*, Admixture proportions

## Abstract

**Ancestry composition** of populations and individuals has been extensively investigated in recent years due to advances in the genotyping and sequencing technologies. As the number of populations and individuals used for ancestry inference increases remarkably, say more than 100 populations or 1000 individuals, it is usually challenging to present the ancestry composition in a traditional way using a rectangular graph. To address this issue, we developed a program, ***AncestryPainter***, which can illustrate the ancestry composition of populations and individuals with a rounded and nice-looking graph to save space. Individuals are depicted as length-fixed bars partitioned into colored segments representing different ancestries, and the population of interest can be highlighted as a pie chart in the center of the circle plot. In addition, *AncestryPainter* can also be applied to display personal ancestry in a way similar to that for displaying population ancestry. *AncestryPainter* is publicly available at http://www.picb.ac.cn/PGG/resource.php.

## Introduction

Ancestry composition of individuals indicates the average proportion of contributing ancestries to their entire genomes [1]. Global ancestry for individuals is frequently inferred in population genetics to understand the genetic relationship and history of gene flow of populations [2], [3], figure out ancestry composition of admixed populations [4], [5], [6], or detect population stratification [7], [8]. Ancestry of a population or an individual can be estimated in different ways, through simple calculation based on allele frequency of the ancestral source populations with K-mean clustering, or using model-based methods such as *ADMIXTURE* [1], *structure* [9], and *frappe* [2], [10]. To use these methods, it is assumed that the observed individuals are drawn from a population with contributions from *K* ancestries. Then the programs estimate the ancestry proportions contributed by *K* ancestral source populations, where *K* is a potential number of ancestral source populations. In theory, the sum of the ancestry proportions of each individual attributed to *K* ancestral source populations is expected to be 100%. However, it does not necessarily mean that all of the ancestral source populations truly contribute ancestry to the target individual or population and statistical tests can be applied to estimate the significance of ancestry contributions.

**Figure 1:**
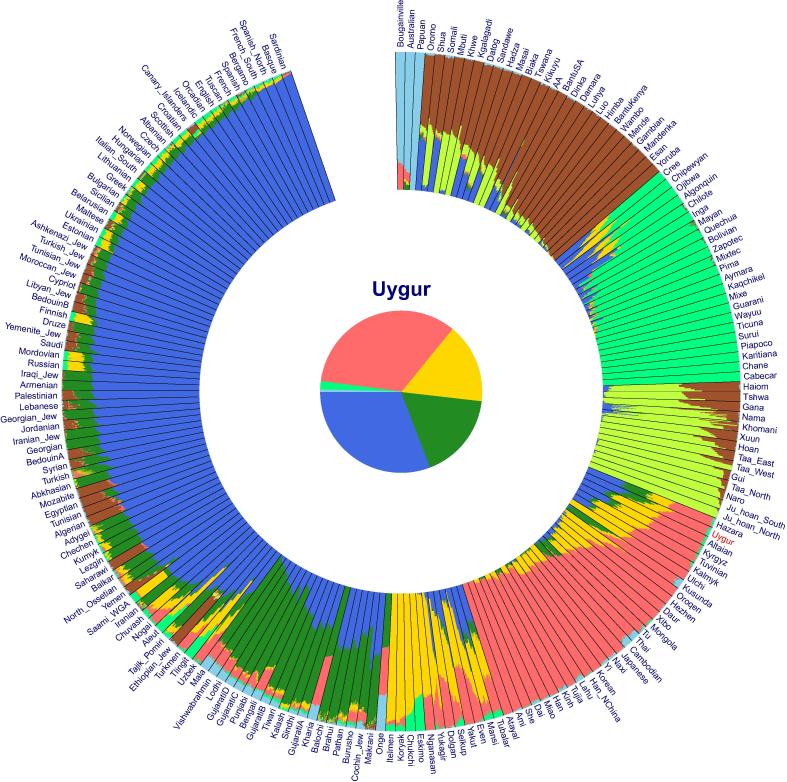
**An example graph taken from the output of *AncestryPainter*** The input of this figure is produced by running *ADMIXTURE* assuming eight ancestral source populations (*K* = 8) based on the Human Origins datasets [12] including Africans, Americans, Oceanians, West Eurasians, South Asians, Central Asians/Siberians, and East Asians. The Uyghur population is highlighted in the center of the graph. The command line used for this result is “perl AncestryPainter.pl -i data.ind -q data.Q -t Uygur”. This example is also included in the *AncestryPainter* program package, which can be downloaded at http://www.picb.ac.cn/PGG/resource.php.

Several computational tools are available to graphically display the estimated ancestry proportions of individuals or populations [9], [11]. These programs display the ancestry of an individual as a fixed-length line segment partitioned into *K* colored components, with the length of each colored component corresponding to the proportion of each ancestry. However, all these programs align individuals in a rectangular graph, which is not able to accommodate a large number of individuals or populations in a single print page, thus making it difficult to publish these results. To solve this problem, we developed *AncestryPainter*, a computational program for displaying the ancestry composition of individuals and populations in a circular graph, and highlighting the ancestry composition of individuals that are of particular interest to the users in the center of the circle as a pie chart (Figure 1). Moreover, *AncestryPainter* automatically aligns populations based on the proportions of their representative ancestries, *i.e.*, the ancestry accounting for the largest proportion in the population. Therefore, the graph generated by *AncestryPainter* is not only nice-looking, but also efficient in ancestry visualization.

## Method

The graphic program of *AncestryPainter* is written in Perl. By running the Perl program, an R script is produced based on the input ancestry composition of each population and then automatically run to plot the figure. The final figure is a circle with fixed perimeter and radius by automatically adjusting the relative space of each input population. It is of note that in *AncestryPainter*, for the purpose of presentation, same space is allocated to each population, regardless of the number of individuals included in each population.

*AncestryPainter* automatically sorts the populations/individuals according to ancestry proportions when displaying the figure. Firstly, the ancestry that accounts for the largest proportion in the population is determined (defined as the representative ancestry of the population) and the input populations are categorized into *K* groups accordingly. Then, populations in each of the *K* groups are plotted in a descending order of the representative ancestry proportions. Individuals within each population are also sorted based on a descending order of its representative ancestry proportion.

## Implementation

*AncestryPainter* requires users to provide a matrix of ancestry proportions of each individual as input data, which can be obtained from any analysis of personal or population ancestry. The program also requires a predefined group identifier of each individual to classify and label the individuals in the graph. The predefined group identifier could be population label, sampling location, or phenotypic classification. For instance, *ADMIXTURE* output file [1] that includes a matrix of ancestry proportions (.Q file) can be used directly as the input data of *AncestryPainter*. In theory, ancestry proportions obtained using any approach could be provided as the input data for *AncestryPainter* as long as the proportions across ancestral populations sum to one, and the input format is modified to meet software requirements. Users can run *AncestryPainter* with a command line under Linux, Mac, or Windows operating systems. It produces and automatically runs an R script to plot a figure file for displaying personal or population ancestry composition. The output of *AncestryPainter* includes a figure (pdf or png), the R script, the color scheme used for plotting the figure, and a file containing the ancestry proportions as well as population label of each individual.

## Display options and features

Figure 1 exemplifies the display for the ancestry composition of global populations using *AncestryPainter* with the Uyghur population highlighted in the center. Dataset used to generate Figure 1 included 2345 individuals representing 203 populations from the Human Origins dataset [12]. The ancestry proportions of each population were obtained using *ADMIXTURE* program, assuming 8 pseudo ancestries (*K* = 8). Default color scheme was used in the figure and populations were automatically sorted according to admixture proportions. As shown in Figure 1, Uyghurs are composed of four major ancestries, with representative ancestral source populations from West Eurasia, South Asia, East Asia, and Siberia, respectively. *AncestryPainter* allows users to freely modify the default settings such as the color scheme, the order of populations/individuals, and what populations/individuals to be included with command options. Users can also specify a certain population or individual to be highlighted in the center of the graph, or plot without any population or individual highlighted in the center. If there are too many populations to present, users can remove the black lines between populations for a better display.

In addition to displaying ancestry composition of individuals, *AncestryPainter* could be applied more extensively to illustrate any data that can be presented in a bar plot. As long as the assigned proportions for all items sum to 100%, the data can be displayed in a circular graph in the same way as shown for the personal or population ancestry composition.

## Future developments

In this study, we developed *AncestryPainter*, a computational program that can be used to illustrate the ancestry compositions of populations and individuals with a rounded and nice-looking graph in a spatially-efficient way. In the future, we will implement more functions, for instance, to allow displaying ancestry compositions of populations assuming different number of ancestral populations (multiple *K*s) in one image using multiple concentric circles, so that ancestry compositions at different levels could be presented in an explicit and efficient way.

## Authors’ contributions

SX conceived and designed the study. DL and QF developed the program and wrote the computer code. QF drafted the manuscript and SX revised the manuscript. All authors read and approved the final manuscript.

## Competing interests

The authors declare that they have no competing interests.
